# Effects of Resistance to Bt Cotton on Diapause in the Pink Bollworm, *Pectinophora gossypiella*

**DOI:** 10.1673/031.007.4901

**Published:** 2007-09-18

**Authors:** Yves Carrière, Christa Ellers-Kirk, Robert W. Biggs, Maria A. Sims, Timothy J. Dennehy, Bruce E. Tabashnik

**Affiliations:** ^1^ Department of Entomology, The University of Arizona, 410 Forbes Building, Tucson, AZ 85721-0036; ^2^ Department of Entomology, The University of Arizona, 37860 W. Smith-Enke Road, Maricopa, AZ 85239

**Keywords:** fitness costs, *Bacillus thuringiensis*, pleiotropic effects

## Abstract

Fitness costs associated with resistance to *Bacillus thuringiensis* (Bt) crops are expected to delay the evolution of resistance. In a previous study where pink bollworm, *Pectinophora gossypiella* (Lepidoptera: Gelechiidae), larvae overwintered in outdoor insectaries, individuals from Bt-resistant strains had lower survival than individuals from Bt-susceptible strains or F1 progeny from crosses between resistant and susceptible adults. To investigate the physiological basis of such recessive cost, diapause duration was experimentally manipulated in the laboratory. Compared to a Bt-susceptible strain and F1 progeny, we hypothesized that Bt-resistant strains could exhibit a lower propensity or intensity of diapause, faster weight loss during overwintering, lower initial weight of diapausing larvae, and reduced longevity of moths emerging from diapause. Results were as expected for initial weight of diapausing larvae and longevity of overwintered male moths or female moths remaining in diapause for a short period. However, a higher diapause induction and intensity and slower weight loss occurred in F1 progeny and Bt-resistant strains than in a Bt-susceptible strain. Moreover, F1 progeny had greater overwintering survival than the Bt-resistant and Bt-susceptible strains, and F1 female moths had the greatest longevity after sustaining long diapausing periods. All of these unexpected results may be explained by pleiotropic effects of resistance to Bt cotton that increased the strength of diapause in the F1 progeny and Bt-resistant strains. Incomplete resistance was reflected in disadvantages suffered by Bt-resistant individuals feeding on a Bt diet instead of a non-Bt diet, including lower diapause propensity, lower diapause intensity and reduced longevity of overwintered male moths. While this study suggests that the evolution of resistance to Bt cotton and feeding on a Bt diet in Bt-resistant individuals have pervasive effects on several traits associated with diapause, further field experiments are needed to elucidate the basis of the overwintering cost in the pink bollworm.

## Introduction

Transgenic cotton producing *Bacillus thuringiensis* (Bt) toxins is increasingly used worldwide for the control of key lepidopteran pests (James 2004; Lawrence 2005). When considered within broad risk assessment and integrated pest management (IPM) systems, Bt cotton may provide environmental and agronomic benefits such as a reduction in application of broad-spectrum insecticides and yield improvements (Carpenter et al. 2002; Capalbo et al. 2003; Snow et al. 2005; Cattaneo et al. 2006). However, the evolution of resistance by target pests is considered a threat to the continued success of Bt crops (Gould 1998; Tabashnik et al. 2003, 2006).

The refuge strategy is used in the USA and elsewhere to delay the evolution of resistance to Bt cotton. The strategy consists of growing non-Bt cotton near Bt cotton to promote mating between susceptible and resistant insects (U.S. EPA 2001). Because resistance is often recessive in target pests, such matings are expected to reduce the heritability of resistance and slow the evolution of resistance (Gould 1998; Carrière et al. 2004a; Tabashnik and Carrière 2007). In addition to refuges, fitness costs associated with resistance to Bt are expected to delay the evolution of resistance (Carrière and Tabashnik 2001; Carrière et al. 2004a; Tabashnik et al. 2005a). Costs are manifested as lower fitness in resistant compared to susceptible insects in the absence of Bt toxins. Costs associated with resistance to Bt are common (Groeters et al. 1994; Carrière et al. 2001a, b; Bird and Akhurst 2005; Higginson et al. 2005; Janmaat and Myers 2005; Gassman et al. 2006) but knowledge of their physiological basis remains limited.

Although several mechanisms can confer resistance to Bt (e.g., reduction in toxin activation by proteases, increased immune function), resistance often involves reduced binding of Bt toxins to midgut membrane receptors (Ferré and Van Rie 2002; Griffitts and Aroian 2005). Information on the molecular basis of reduced binding is available in four lepidopteran cotton pests. In three species including the pink bollworm, *Pectinophora gossypiella* (Saunders), resistance is tightly linked to mutations at a gene encoding a cadherin protein (Gahan et al. 2001; Morin et al. 2003; Xu et al. 2005). In the beet armyworm, *Spodoptera exigua* (Hübner), a Bt resistant strain lacks an aminopeptidase N receptor (Herrero et al. 2005).

Cadherin proteins in the insect midgut membrane may contribute to cell-cell adhesion (Gessner and Tauber 2000; Aimanova et al. 2006). Thus, fitness costs of Bt resistance could arise because cadherin mutations increase permeability of the gut membrane to toxic phytochemicals (Carrière et al. 2004a). Costs were greater in *P. gossypiella* fed a diet with gossypol than a diet without gossypol, which supports the increased permeability hypothesis (Carrière et al. 2004b). Costs could also occur if modification of receptors reduces food assimilation rates (Carrière et al. 2004a, b) or increases energy metabolism due to faster replacement of midgut cells in resistant than susceptible insects (Dingha et al. 2004). An experiment assessing the increased metabolism hypothesis did not find a difference in metabolic rate between a Bt-resistant and Bt-susceptible strain of the beet armyworm, *Spodoptera exigua*, fed on non-Bt diet (Dingha et al. 2004).

In a previous experiment, resistance to Bt cotton was associated with lower overwintering survival in *P. gossypiella* (Carrière et al. 2001a). The goal of this study was to investigate the physiological basis of this cost. We hypothesized that the overwintering cost in resistant versus susceptible individuals could be due to greater depletion of resources during diapause, lower initial diapausing weight, or disruption of the timing of diapause induction or termination. To test these hypotheses, related resistant and susceptible strains and their F1 progeny were compared in terms of diapause induction, rate of weight loss during diapause (an index of metabolic rate), initial weight of diapausing larvae, diapause intensity, overwintering survival, and longevity of overwintered moths.

## Materials and Methods

### *P. gossypiella* diapause

*P. gossypiella* overwinters as fourth instar larvae in cotton bolls, ground litter or in the soil (Bariola and Henneberry 1980; Henneberry and Jech 1999). Diapause is induced by short day length (< 13 hours) and favored by cool temperatures (Bull and Adkisson 1960; Ankersmit and Adkisson 1967). Other factors such as nutrition and moisture can modulate diapause induction and termination (Bull and Adkisson 1960; Watson et al. 1973). After resuming development, diapausing larvae metamorphose into pupae and emerge as moths soon after. The temperature threshold for development is 13°C (Watson et al. 1973; Carrière et al. 2001c).

*P. gossypiella* was introduced to the southwestern United States at the beginning of the 20^th^ century (Henneberry and Naranjo 1998). Despite arriving in Arizona at least 80 years ago (Henneberry and Naranjo 1998), this pest still has maladaptive early emergence from diapause called “suicidal emergence,” which is emergence and death of moths up to several months before phenologically suitable cotton is available (Henneberry and Jech 1999; Carrière et al. 2001c).

### *P. gossypiella* strains

Three strains of *P. gossypiella* were reared on wheat germ diet: an unselected parent strain (MOV97) and two resistant strains derived from the parent strain and exposed repeatedly to Cry1Ac in diet (MOV97-R_10_ and MOV97-R_100_) (Carrière et al. 2001a, 2004b). Bt cotton produces Bt toxin Cry1Ac, which kills larvae of some lepidopteran pests. Also tested were F1 progeny from reciprocal crosses between each of the resistant strains and their unselected parent strain (100 MOV97-R_10_ males × 100 MOV97 females and vice versa; 50 MOV97-R_100_ males × 50 MOV97 females and vice versa). MOV97 was started from *P. gossypiella* collected in the Mohave Valley of western Arizona in 1997 and was not exposed to toxin in the laboratory (Tabashnik et al. 2000). When this study was started, the number of laboratory-reared generations was 34 for MOV97, 21 for MOV97-R_10_ after its derivation from the F10 generation of MOV97, and 4 for MOV97-R_100_ after its derivation from the F28 generation of MOV97-R_10_ (Carrière et al. 2001a, 2004b). MOV97-R_10_ had been selected 11 times with 10 µg of Cry1Ac per ml of diet. MOV97-R_100_ had been selected nine times with 10 µg of Cry1Ac per ml of diet (while still part of MOV97-R_10_) and twice with 100 µg of Cry1Ac per ml of diet (after splitting from MOV97-R_10_). Homozygous resistant individuals derived from MOV97 survived on Bt cotton (Tabashnik et al. 2005b; Carrière et al. 2006).

To estimate resistance allele frequency, neonates were fed diet with a diagnostic toxin concentration (10 µg Cry1Ac per ml of diet) that allows survival only of homozygous resistant individuals (Tabashnik et al. 2000). Resistance allele frequency was estimated as the square root of adjusted proportion survival (survival at the diagnostic concentration divided by survival on untreated diet). When MOV97 was started from field-collected insects in 1997, its estimated resistance allele frequency was 0.47 (Tabashnik et al. 2000). Immediately before the experiment, estimated resistance allele frequencies were 0.16 for MOV97, 0.96 for MOV97-R_10_, 1.0 for MOV97-R_100_, 0.59 for MOV97 × MOV97-R_10_, and 0.51 for MOV97 × MOV97-R_100_.

### Experiment 1: Effect of strain and diet on induction of diapause, the rate of weight loss during diapause, and the initial weight of diapausing larvae

For each strain and set of F1 progeny, 100 neonates were placed individually in cups (28 ml) containing untreated diet. For the MOV97-R_10_ and MOV97-R_100_ strains, 100 neonates were also placed individually in cups with treated diet (10 µg Cry1Ac per ml of diet). Cups were placed under diapausing conditions (25°C photophase/15°C scotophase, 11Lr:13D, 30% rH) for eight weeks, at which time all insects had developed to at least the 4^th^ instar (i.e., the diapausing instar). Individual fourth instars were placed between two filter papers in 100 × 15 mm Petri dishes and returned to diapausing conditions for 12 days. After 12 days, insects were washed with distilled water, sexed (based on presence of gonads in males), weighed, and returned to clean Petri dishes between two filter papers. Petri dishes were kept for two more days under diapausing conditions and transferred to overwintering conditions (20°C photophase/7°C scotophase, 10L:14D, 100% rH). While under overwintering conditions, insects were weighed every 4 weeks for 28 weeks to estimate weight loss.

### Statistical analysis

Pearson χ^2^ tests were used to compare diapause proportion between MOV97 and the two sets of F1 progeny on non-Bt diet, MOV97 and the two resistant strains on non-Bt diet, and between MOV97-R_10_ and MOV97-R_100_ on non-Bt and Bt diet. Weight loss was estimated for each individual from the slope of a linear regression between weight and time in diapause. The absolute value of the slopes was used as an estimate of weight loss (i.e., higher absolute values show faster weight loss). Weight loss was estimated only for insects weighed four times or more. Multiple regression was used to assess the effect of strain/diet (considered as a single categorical factor), sex, initial weight, and the interaction between these factors on weight loss. Linear contrasts of least squares means were used to further assess the effects of strain/diet on weight loss. Multiple regression was used to assess the effect of strain/diet, sex, and the interaction between these factors on initial weight of the diapausing larvae. Linear contrasts of least squares means were used to further assess the effects of strain/diet on initial diapausing weight.

**Table 1.**
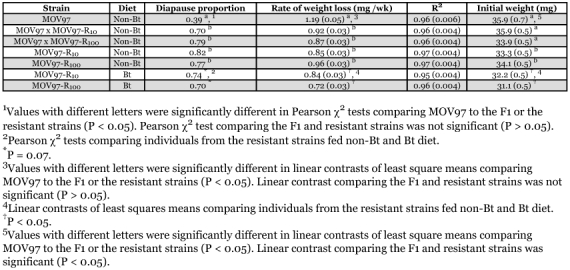
Effect of strain and diet on diapause proportion, rate of weight loss during overwintering, and initial weight of diapausing larvae (least squares means ± SE from multiple regression models are shown for last two variables, see text). Also shown is the average R^2^ (plusmn; SE) of slopes used to estimate weight loss.

### Experiment 2: Effect of strain and diet on diapause intensity, overwintering survival, and longevity of overwintered adults

For each strain and each set of F1 progeny, 100 neonate larvae were transferred to each of 20 cups (450 ml) containing non-Bt diet (21 cups for MOV97-R_10_). 100 neonates from MOV97-R_10_ were also transferred to 20 cups with Bt diet (10 µg Cry1Ac per ml of diet). Cups were maintained under diapause-inducing conditions as above. After six weeks, fourth instars were removed from cups and put between two pieces of filter paper in Petri dishes (150 diameter × 15 mm height). Each Petri dish contained ca. 20 larvae and a 1 cm^2^ piece of non-Bt diet. After an additional 10 days under diapause-inducing conditions, fourth instars were sexed and moved to new Petri dishes containing only 10 males or 10 females between pieces of filter paper (i.e., no diet). The Petri dishes were returned to diapause-inducing conditions for 8 more days, after which any pupae, moths, or dead insects were removed. The remaining fourth instars, which were mainly found in hibernacula, were considered in diapause.

Petri dishes with diapausing larvae were transferred to overwintering conditions as above. After spending 8, 16, 20 or 32 weeks under overwintering conditions, a subset of Petri dishes were transferred to diapause-breaking conditions (constant 22°C, 14L:10D, 30% rH). An equal number of dishes containing males or females were transferred for each strain and each set of F1 progeny on each date. The filter papers were misted with distilled water after transfer to diapause-breaking conditions and twice a week thereafter to break diapause (Watson et al. 1973). Newly formed pupae were transferred individually to 28-ml cups with moist paper towels and kept until moth emergence. Moths were kept individually in 28-ml cups with access to honey water and adult longevity was recorded for insects transferred after 8, 16 or 20 weeks.

### Statistical analyses

Some moths were found when Petri dishes were transferred to diapause-breaking conditions, showing that some larvae broke diapause while under overwintering conditions. Hereafter, we call this “winter emergence,” which indicates a low intensity of diapause. The proportion of winter emergence was calculated for each Petri dish as the number of moths emerging under overwintering conditions divided by the number of insects in diapause at the time of transfer to overwintering conditions. To assess among-strain differences in the proportion of winter emergence, multiple regression was used to evaluate the effects of strain/diet, number of weeks in diapausing conditions, sex, and the interactions between these factors. The experimental unit in this analysis is a Petri dish. A multiple regression model with the same explanatory variables was used to assess factors affecting the proportion of overwintering survival and moth longevity. The proportion of overwintering survival was calculated for each Petri dish as the number of moths emerging under diapausing-breaking conditions divided by the number of larvae still in diapause when transferred to diapausing-breaking conditions. Linear contrasts on least squares means were used in all cases to further investigate the effects of strain/diet on the response variables.

**Figure 1.**
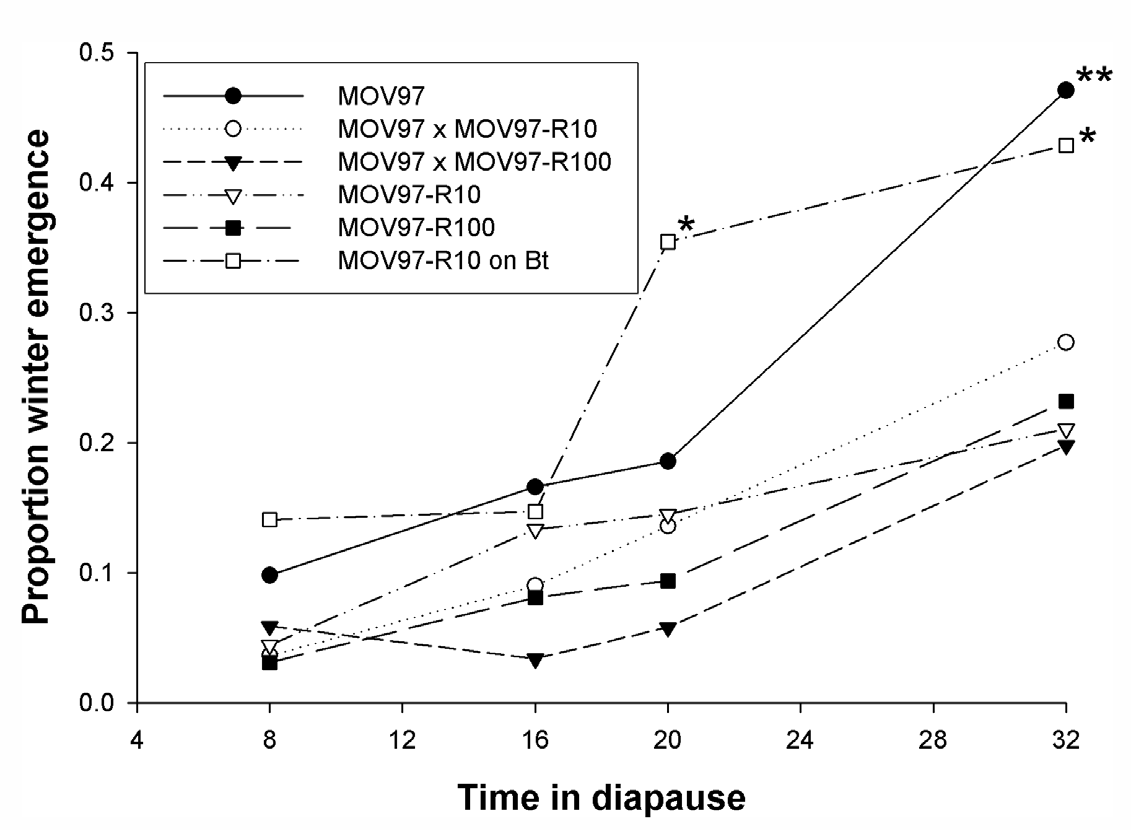
Effect of time in diapause and diet (combined) on the proportion of winter emergence (least squares means from multiple regression model, see text). Standard errors (not shown) associated with proportions were always equal or smaller than 0.06. * Linear contrast showed a significant difference (P < 0.05) between individuals from MOV97-R_10_ fed Bt and non-Bt diet. ** Linear contrasts showed a significant difference between MOV97 and the F1 progeny or the resistant strains, but no significant differences (P > 0.05) between the F1 progeny and resistant strains.

## Results

### Experiment 1: Effect of strain and diet on induction of diapause, the rate of weight loss during diapause, and initial weight of diapausing larvae

Resistance to Bt cotton was associated with higher diapause induction (i.e., an increase in the proportion of diapause), slower weight loss during diapause, and lower initial weight of diapausing larvae. Moreover, feeding on a Bt diet as opposed to a non-Bt diet by resistant strains reduced the proportion of diapause, weight loss during diapause, and the initial weight of diapausing larvae.

The proportion of diapause was significantly lower in MOV97 than in the F1 progeny and resistant strains (Table 1). However, the proportion of diapause was not significantly different between the resistant strains and the F1 progeny, indicating that the effect of resistance to Bt cotton on diapause induction was dominant. Feeding on a Bt diet instead of a non-Bt diet induced a marginally significant decrease in diapause proportion in the resistant strains (Table 1).

Time spent in diapause was a good predictor of weight loss (R^2^ = 0.95 to 0.97, Table 1). Diapause proportion was negatively correlated with weight loss (least squares means from Table 1 used in analysis, Pearson r = -0.89, P = 0.0071). Sex and the interactions involving Sex had no significant effect on weight loss (Multiple regression, all P values > 0.05). In a model without Sex, a negative association occurred between initial weight of larvae and weight loss (slope = -19.3, df = 1, 459, t = -7.30, P < 0.0001), indicating that larger larvae lost weight at a slower rate than smaller larvae.

**Figure 2.**
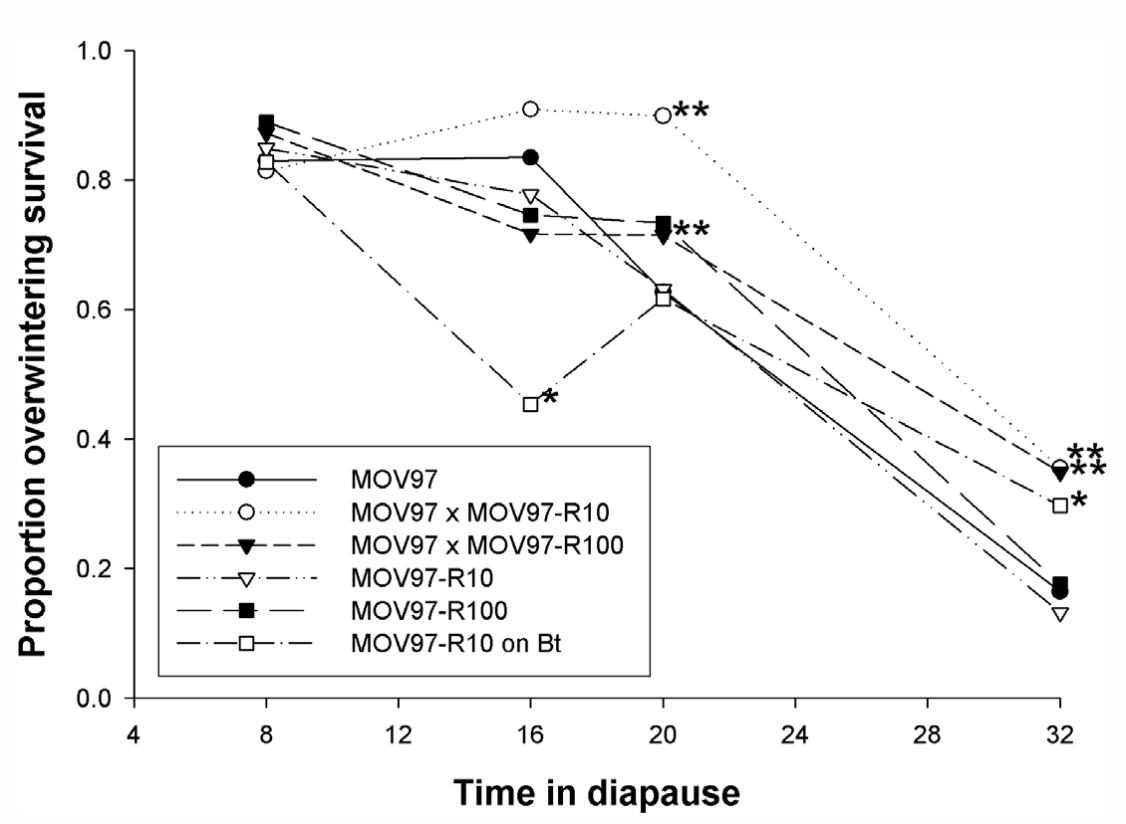
Effect of time in diapause and diet (combined) on the proportion of overwintering survival (least squares means from multiple regression model, see text). Standard errors (not shown) associated with proportions were always equal or smaller than 0.08. * Linear contrast showed a significant difference (P < 0.05) between individuals from MOV97-R_10_ fed a Bt or non-Bt diet. ** Linear contrasts showed a significant difference between the F1 progeny and MOV97 or the resistant strains, but no significant differences (P > 0.05) between MOV97 and the resistant strains.

Independent of the effect of initial larval weight, Strain/diet significantly affected weight loss (df = 6, 459, F = 10.3, P < 0.0001). Linear contrasts revealed that weight loss was significantly lower in the F1 progeny and the resistant strains than in MOV97 (Table 1). However, weight loss was similar in the F1 and resistant strains, indicating that resistance to Bt cotton had a dominant effect on weight loss. Weight loss was significantly lower when individuals from the resistant strains were fed a Bt diet rather than a non-Bt diet (Table 1).

Independent of the effect of Strain/diet, weight loss during overwintering was slower in large than small diapausing larvae. To obtain a better picture of the effect of resistance to Bt cotton on weight loss, we investigated the effect of Strain/diet on the initial weight of diapausing larvae. Sex had similar effects on initial weight within the strains (Sex × Strain/diet interaction, P = 0.46). In a multiple regression model without the Sex × Strain/diet interaction, Strain/diet (df = 6, 459, F = 11.5, P < 0.0001) and Sex (df = 1, 459, F = 115.3, P < 0.0001) significantly affected initial weight. Linear contrasts revealed that initial weight was significantly lower in the resistant strains than in MOV97, although initial weight did not differ between the F1 progeny and MOV97 (Table 1), which indicates that resistance to Bt cotton had a recessive negative effect on the initial weight of diapausing larvae. Initial larval weight was significantly lower when individuals from the resistant strains were fed a Bt diet rather than a non-Bt diet (Table 1).

### Experiment 2: Effect of strain and diet on diapause intensity, overwintering survival, and longevity of overwintered adults

Resistance to Bt cotton was associated with increased diapause intensity (i.e., a reduction in the proportion of winter emergence), increased overwintering survival in F1 progeny, and reduced longevity of overwintered males but variable effects on female longevity. Moreover, feeding on a Bt diet as opposed to a non-Bt diet in MOV97-R_10_ reduced diapause intensity, had variable effects on overwintering survival, and reduced longevity in males remaining in diapause for a long period.

**Table 2.**
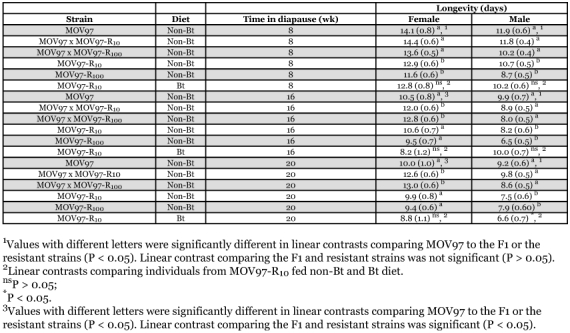
Effect of strain, diet, and time in diapause on longevity of female and male moths emerging from diapause (least squares means ± SE from multiple regression models, see text).

Sex and the interactions involving Sex did not affect the proportion of winter emergence (Multiple regression, all P values > 0.56). In a multiple regression model without Sex, the proportion of winter emergence was affected by Strain/diet (df = 5, 259, F = 7.53, P <0.0001) and time in diapause (df = 3, 259, F= 37.08, P < 0.0001). The interaction between Strain/diet and time was not significant (P = 0.26), suggesting that time in diapause affected winter emergence similarly in all strains. However, linear contrasts revealed no significant differences among strains remaining in diapause for 8 and 16 weeks but significant differences among strains in diapause for 20 and 32 weeks (Figure 1). After 8, 16 and 20 weeks in diapause, winter emergence did not differ significantly between MOV97 and the F1 progeny or the resistant strains. However, after 32 weeks in diapause, winter emergence was significantly greater in MOV97 than in the F1 crosses and the resistant strains, indicating that resistance to Bt cotton resulted in a dominant increase in the intensity of diapause (Figure 1). Moreover, feeding on a Bt diet as opposed to a non-Bt diet increased the proportion of winter emergence in MOV97-R_10_ after 20 and 32 weeks but not in larvae remaining in diapause for 8 or 16 weeks (Figure 1).

Sex and the interactions involving Sex did not affect the proportion of overwintering survival (Multiple regression, all P values > 0.20). In a multiple regression model without Sex, the proportion of overwintering survival was affected by Strain/diet (df = 5, 258, F = 4.83, P = 0.0003), time in diapause (df = 3, 258, F = 164.5, P < 0.0001) and the interaction between these factors (df = 15, 258, F = 2.6, P = 0.0011). Overwintering survival did not differ among MOV97, the F1 progeny and the resistant strains kept in diapause for 8 and 16 weeks (Figure 2). However, overwintering survival was significantly greater in the F1 progeny than in MOV97 after 20 and 32 weeks. No significant differences between MOV97 and the resistant strains were present, showing that resistance to Bt cotton only increased overwintering survival in the F1 progeny. Feeding on a Bt diet had inconsistent effects on overwintering survival in MOV97-R_10_, as survival was reduced after 16 weeks in diapause but increased after 32 weeks (Figure 2).

Sex had a significant effect on adult longevity (df = 1, 1137, F = 113.2, P < 0.0001) and the effect of Strain/diet on adult longevity differed for females and males (Sex × Strain/diet interaction, df = 5, 1137, F = 4.8, P = 0.0002). Thus, variation in adult longevity was analyzed separately for females and males.

In females, Strain/diet (df = 5, 535, F = 12.2, P < 0.0001) and time in diapause (df = 2, 535, F = 27.2, P < 0.0001) significantly affected adult longevity, although the interaction between these factors was not significant (df = 10, 535, F = 1.20, P = 0. 28). Nevertheless, linear contrasts indicated different patterns of longevity in strains remaining in diapause for 8 weeks as opposed to strains remaining in diapause for 16 or 20 weeks. After 8 weeks in diapause, longevity was significantly less in females from the resistant strains than from MOV97 or F1, indicating that a recessive cost affected longevity (Table 2). In contrast, F1 females survived longer than females from MOV97 or the resistant strains after 16 or 20 weeks in diapause (Table 2). Diet (non-Bt or Bt) did not affect female longevity in any of the diapausing periods (Table 2).

In males, Strain/diet (df = 5, 602, F = 9.8, P < 0.0001) and time in diapause (df = 2, 602, F = 32.9, P × 0.0001) had significant effects on longevity. Time in diapause did not affect longevity similarly in the strains (Strain/diet × time in diapause interaction, df = 10, 602, F = 2.06, P = 0.05). Recessive costs affecting longevity were found in all diapausing periods (Table 2). Feeding on a Bt diet in MOV97-R_10_ decreased longevity significantly only for males remaining in diapause for 20 weeks.

## Discussion

When diapause was induced in the laboratory and larvae were allowed to overwinter in the soil in outdoor insectaries, individuals from pink bollworm strains susceptible to Bt cotton and F1 progeny had significantly greater spring emergence than individuals from strains resistant to Bt cotton, which indicated the presence of a recessive overwintering cost (Carrière et al. 2001a). Spring emergence was defined as moths emerging 17–34 weeks after the transfer of diapausing larvae to the field. Here, diapause duration was experimentally manipulated in the laboratory to investigate the physiological basis of such cost. Larvae were transferred to diapause-breaking conditions after 8–32 weeks, which seems approximately comparable to the period preceding spring emergence in an earlier experiment (Carrière et al. 2001a). Compared to a Bt-susceptible strain, we hypothesized that related Bt-resistant strains could exhibit lower induction or intensity of diapause, greater rate of weight loss during overwintering, lower initial weight of diapausing larvae, or a reduction in the longevity of moths emerging from diapause. We did not expect differences between MOV97 and the F1 progeny because the overwintering cost was recessive (Carrière et al. 2001a). Contrary to expectations, a higher proportion of diapause (Table 1), lower weight loss (Table 1), lower proportion of winter emergence (Figure 1), and better overwintering survival (Figure 2) was found in the resistant strains or F1 progeny than in the Bt susceptible strain. In accord with expectations, lower initial weight of diapausing larvae was found in the resistant strains than in the F1 progeny or susceptible strain (Table 1). Male moths from resistant strains, or female moths from resistant strains remaining in diapause for a short period, did have a reduced longevity compared to moths from F1 progeny or the susceptible strain (Table 2). However, female moths from the F1 progeny had the longest longevity after sustaining long periods in diapause. Therefore, this study suggests that the evolution of resistance to Bt cotton has pervasive effects on several traits associated with diapause. However, because the association of resistance with most of these traits did not support our hypotheses, we conclude that the factors underlying the overwintering cost expressed in the field are more complex than envisaged.

The phenotypic effects of feeding on a Bt diet instead of a non-Bt diet was investigated in Bt-resistant individuals. Compared to individuals fed a non-Bt diet, Bt-fed individuals had a lower proportion of diapause (Table 1), a greater proportion of overwintering emergence (Figure 1), lower weight loss during overwintering, and reduced initial diapausing weight (Table 1). Feeding on a Bt diet did not have consistent effects on overwintering survival (Figure 2). In male moths produced from Bt-fed larvae, longevity was reduced only for the longest diapausing period, while diet did not affect longevity in females (Table 2). These results indicate that feeding on a Bt diet in Bt-resistant individuals could have negative consequences on fitness, occurring mainly through a reduction in diapause propensity and intensity and negative effects on longevity of overwintered male moths. Such reduced fitness is called incomplete resistance, which involves disadvantages suffered by Bt-resistant insects on a Bt crop relative to a non-Bt crop (Liu et al. 2001; Bird and Akhurst 2004). Feeding on a Bt diet as opposed to a non-Bt diet increased metabolic rate in non-diapausing Bt-resistant beet armyworm larvae, indicating that replacement of midgut cells was increased by damages caused by Bt toxins (Dingha et al. 2004). While these results are opposite to the reduced weight loss (indicative of lower metabolism) observed here (Table 1, resistant strains on Bt diet), the studies are not directly comparable because midgut cell replacement is probably much slowed during pink bollworm diapause.

Much of the divergence among strains fed a non-Bt diet measured in this study may have been driven by variation in the strength of diapause. The lower proportion of diapause (Table 1) and higher proportion of winter emergence (Figure 1) in MOV97 than in the F1 progeny and resistant strains indicate that the strength of diapause was increased in individuals bearing one or more resistance alleles. Diapause is a state of quiescence typically associated with a reduction of metabolic rate and depletion of resources needed to survive harsh conditions (Morris and Felton 1970; Irwin and Lee 2000; Ellers and Van Alphen 2002). Thus, the greater strength of diapause in the F1 progeny and resistant strains was associated with lower weight loss during diapause in these strains (Table 1). Such among-strain variation in weight loss would be expected to confer an overwintering survival advantage to individuals from the F1 progeny and resistant strains over individuals from MOV97. However, large individuals have better diapausing survival than small individuals (Morris and Felton 1970; Ellers and Van Alphen 2002), not only because larger individuals may have more reserves, but possibly because their rate of weight loss during diapause is also reduced, as shown here. Thus, because initial diapausing weight was significantly lower in the resistant strains than in the F1 progeny and MOV97 (Table 1), one would expect the among-strain variation in diapausing weight to contribute to a reduction of survival in the resistant strains. Accordingly, the greater overwintering survival in the F1 progeny than in MOV97 and the resistant strains (Figure 2) can be explained by combining the effects of the among-strain variation in weight loss (favoring the F1 progeny and resistant strains) and in initial diapausing weight (disfavoring the resistant strains). Finally, variation in longevity was much like variation in initial larval diapausing weight in males, and in females spending a short period in diapause (compare Table 1 and Table 2). However, as for variation in overwintering survival, variation in longevity of females emerging after longer diapausing periods (Table 2) may have resulted from the combined effects of variation in initial diapausing weight and weight loss. Because males and females often need different levels of energy reserves to sustain adult fitness (Blackenhorn et al. 1995, Presiozi and Fairbairn 2000), the consequence of variation in initial larval weight and weight loss on longevity does not need to be the same in both sexes.

It remains possible that the divergence between strains was due to factors unrelated to resistance, such as variation in genetic background resulting from genetic drift. MOV97-R_10_ had been separated from MOV97 for 10 generations when costs were investigated in larvae overwintering in the field (Carrière et al. 2001a). MOV97-R_10_ and MOV97-R_100_ had been separated from MOV97 for 21 generations at the time of this experiment, which could have favored drift. However, susceptible and resistant strains were maintained at >200–1000 adults per generation, which is expected to reduce the effects of drift (Falconer 1981). Moreover, much of the variation among strains fed a non-Bt diet can be traced to a divergence in the strength of diapause (see above), which may well have resulted from pleiotropic effects of the cadherin mutations conferring resistance to Bt cotton.

Changes in diapause resulting from the disruptive effects of resistance have been documented in at least three lepidopteran species. In the codling moth, *Cydia pomonella* (Linnaeus), resistance to insecticides involved a reduction in development rate due to pleiotropic effects of the resistance alleles (Boivin et al. 2003). The slower development in resistant compared to susceptible individuals delayed exposure of the diapause-sensitive stage to the critical photoperiod inducing diapause, which resulted in resistant individuals completing a lower number of generations in the field than susceptible individuals. A higher critical photoperiod for diapause induction was also found in resistant strains compared to heterozygous or susceptible strains, which partly explained the earliest induction of diapause in resistant individuals (Boivin et al. 2004). In the Obliquebanded leafroller, *Choristoneura rosaceana* (Harris), resistance to insecticides was associated with a higher propensity of estival diapause (Carrière et al. 1995). Changes in the additive genetic variance and heritability of diapause propensity, as well as in the genetic correlation between diapause propensity and development rate, supported the hypothesis that the spread of resistance alleles had strong pleiotropic effects on diapause (Carrière and Roff 1995). On the other hand, increased resistance to Bt cotton in *Helicoverpa armigera* (Hübner) was not associated with greater pupal diapause (Bird and Akhurst 2004). Nevertheless, diapausing pupal weight and overwintering survival was greater in a susceptible strain than in related heterozygous and resistant strains, suggesting that resistance to Bt cotton induced a recessive cost affecting diapause in *H. armigera*.

We do not know why no cost affecting overwintering survival was seen in the present experiment. Part of the answer may be that the low rates of overwintering weight loss observed in the F1 progeny and resistant strains (Table 1) are advantageous in the laboratory but detrimental in the field. Activation of immune defenses to combat parasites may require a high metabolic rate (Schmid-Hempel 2005). For example, diapausing pupae of the white cabbage butterfly, *Pieris brassicae* (Linnaeus), increased their metabolic rate by 8% when submitted to an immune challenge (Freitak et al. 2003). The warm weather occurring during the diapausing period of the pink bollworm may allow frequent threats of diapausing individuals by parasites (e.g., fungus, nematodes, etc.). If a low metabolic rate reduced the response to immune challenges, then the overwintering advantage conferred by low metabolism could be overridden by greater mortality induced by parasites, thereby resulting in recessive overwintering costs in the field. Moreover, this ecological trade-off could be involved in the persistence of maladaptive early spring emergence in *P. gossypiella* (see Carrière et al. 2001c), because the evolution of a low metabolic rate required for postponing the termination of diapause would be blocked by a concomitant increase in mortality from parasites. Thus, a study of the interactions between resistance to Bt cotton, metabolic rate, and susceptibility to parasites could provide a basis to better understand the nature of overwintering costs and address the conundrum of the persistence of suicidal emergence in the pink bollworm.
